# In-hospital SGLT2 inhibitor initiation, prescribing gaps, and 30-day all-cause readmission in heart failure with reduced ejection fraction: a US post-guideline cohort study

**DOI:** 10.1186/s12872-026-06184-y

**Published:** 2026-06-30

**Authors:** Otabek Pulatov, Soo Young Kim, Zvi Grossman, Farhan Noor, Bilal Salam, Tahmid Khan, Akhila Matam, Shan Wang, Thomas Caraccio, Kevin P. Marzo

**Affiliations:** 1 NYU Grossman Long Island School of Medicine, 101 Mineola Boulevard, Mineola, NY 11501 USA; 2 Department of Biostatistics, NYU Grossman Long Island School of Medicine, Mineola, NY USA; 3 Department of Pharmacy, NYU Langone Hospital—Long Island, Mineola, NY USA; 4 Department of Medicine, Division of Cardiology, NYU Langone Health— Long Island, Mineola, NY USA

**Keywords:** SGLT2 inhibitors, Heart failure with reduced ejection fraction, Hospital readmission, Guideline-directed medical therapy, Propensity score, Quality improvement

## Abstract

**Background:**

Heart failure accounts for more than one million US hospitalizations annually, with 30-day all-cause readmission approaching 25% and triggering CMS Hospital Readmissions Reduction Program penalties. The 2022 ACC/AHA/HFSA guideline and the 2023 ESC focused update elevated SGLT2 inhibitors to Class I therapy for heart failure with reduced ejection fraction (HFrEF) [1, 2]. The DAPA ACT HF–TIMI 68 prespecified meta-analysis demonstrated reductions in cardiovascular death or worsening heart failure (HR 0.71) and all-cause mortality (HR 0.57).

Real-world prescribing patterns and 30-day readmission outcomes in the post-guideline US era are not well characterized. The relative contribution of clinical stability variables versus co-prescribed guideline-directed medical therapy (GDMT) to confounding has not been directly quantified in this setting.

**Methods:**

We conducted a retrospective cohort study at four NYU Langone Health hospitals from January 2023 to January 2026. Adults with a primary heart failure discharge diagnosis were included.

The prespecified primary analysis was in the HFrEF subgroup (LVEF ≤ 40%). The primary outcome was 30-day all-cause readmission. Stabilized inverse probability of treatment weighting (IPTW) was the primary adjustment, with overlap weighting (ATO) as sensitivity analysis.

Hierarchical logistic regression decomposed the confounding contribution of clinical stability parameters relative to GDMT. The E-value assessed robustness to unmeasured confounding.

**Results:**

Among 438 patients, 122 (27.9%) received in-hospital SGLT2 inhibitor initiation. The HFrEF rate was 41.6%, a sixfold increase from 6.6% reported in INSIGHT-HF (2020–2021). Patients with prior heart failure hospitalization received SGLT2 inhibitors at 11.4% versus 29.7% in those without (*p* < 0.001). In HFrEF (*n* = 221), 30-day readmission was 12.1% versus 31.8% (crude OR 0.29, 95% CI 0.14–0.61). The primary IPTW estimate was OR 0.34 (95% CI 0.13–0.91, *p* = 0.032). Sensitivity analyses were directionally consistent. Clinical stability parameters contributed only 9.3% confounding attenuation; GDMT was the dominant confounder.

**Conclusions:**

In a contemporary US post-guideline cohort, in-hospital SGLT2 inhibitor initiation reached 41.6% in HFrEF but remained low in patients with recent heart failure hospitalization. In-hospital SGLT2 inhibitor initiation was associated with lower 30-day all-cause readmission, though initiation was strongly bundled with discharge GDMT optimization and cannot be distinguished from a GDMT optimization effect with this study design. These findings should be considered hypothesis-generating. Because short-term safety events and post-discharge persistence were not systematically captured, these findings should not be interpreted as establishing the benefit-risk profile of inpatient SGLT2 inhibitor initiation. The prescribing gap in high-risk patients is an actionable quality-improvement target.

**Supplementary Information:**

The online version contains supplementary material available at https://doi.org/10.1186/s12872-026-06184-y.

## Introduction

Heart failure accounts for more than one million hospitalizations annually in the United States, with 30-day readmission rates approaching 25% despite decades of quality-improvement effort [3].

The CMS Hospital Readmissions Reduction Program (HRRP), enacted in 2012, penalizes hospitals for excess heart failure readmissions. National rates have plateaued rather than declined [4]. This plateau persists despite four pillars of guideline-directed medical therapy (GDMT) now carrying Class I recommendations for heart failure with reduced ejection fraction (HFrEF) [1].

Sodium-glucose cotransporter 2 (SGLT2) inhibitors occupy a distinctive position among GDMT.

They have a favorable initiation profile in the acute setting, with minimal blood pressure effects, fixed dosing, and natriuretic properties [5]. DAPA-HF and EMPEROR-Reduced established benefits in chronic HFrEF regardless of diabetes status [6, 7]. EMPULSE and SOLOIST-WHF demonstrated safety and efficacy when initiation occurs in or near hospitalization [8, 9]. The DAPA ACT HF–TIMI 68 trial showed a neutral 2-month primary outcome, but the prespecified three-trial meta-analysis demonstrated reductions in cardiovascular death or worsening heart failure (HR 0.71, 95% CI 0.54–0.93) and all-cause mortality (HR 0.57, 95% CI 0.41–0.80) [10]. The 2022 ACC/AHA/HFSA guideline elevated SGLT2 inhibitors to Class I therapy [1]. INSIGHT-HF reported a 6.6% in-hospital initiation rate during 2020–2021, before the guideline update [11].

Three gaps in the existing evidence base motivate this study. First, no completed clinical trial has used 30-day all-cause readmission — the HRRP penalty metric — as a primary endpoint.

Second, prior observational studies have relied on administrative claims data lacking clinical parameters [12], or used propensity matching with few covariates and without GDMT adjustment [13].

The relative confounding contribution of clinical stability parameters versus GDMT in this setting has not been directly quantified. Third, and most importantly, the prescribing landscape has shifted considerably since these earlier studies. A contemporary US post-2022 guideline cohort captures a different prescribing environment — one with higher SGLT2 inhibitor uptake, evolved patient selection, and greater concurrent GDMT intensity — that earlier implementation analyses were not designed to capture.

We conducted a retrospective cohort study using granular electronic health record data from a large US academic health system to address these gaps. The primary objective was to assess the association between in-hospital SGLT2 inhibitor initiation and 30-day all-cause readmission, with the prespecified primary analysis in HFrEF where biological rationale and guideline evidence are strongest. The methodological objective was to quantify the confounding contribution of clinical stability parameters absent from prior claims-based studies.

## Methods

### Study design and setting

This was a retrospective cohort study at NYU Langone Health, a US academic health system with four acute-care hospitals: Tisch Hospital (Manhattan), Hospital—Long Island (Mineola), Hospital—Brooklyn, and Hospital—Suffolk. The Epic-based electronic health record provided vital signs, laboratory values, medications, and echocardiographic data unavailable in administrative claims. The study period was January 2023 through January 2026, capturing the post-2022 ACC/AHA/HFSA guideline era. The Institutional Review Board approved the study with waiver of informed consent (Protocol s25-00254). Reporting followed STROBE guidelines [14].

### Study population

We identified adults (≥ 18 years) with a primary heart failure discharge diagnosis (ICD-10-CM I50.x). Left ventricular ejection fraction (LVEF) was ascertained from transthoracic echocardiography during the index admission or within the prior 12 months. HFrEF was defined as LVEF ≤ 40%.

Exclusion criteria were SGLT2 inhibitor use within 12 months before admission; type 1 diabetes mellitus; history of diabetic ketoacidosis; end-stage renal disease or chronic dialysis; in-hospital death; discharge to hospice facility; missing LVEF; and discharge disposition recorded as “Still a Patient.” Only the first qualifying admission was included for patients with multiple eligible admissions. The final analytic cohort comprised 438 patients (122 SGLT2 inhibitor recipients, 316 controls).

### Exposure

SGLT2 inhibitor exposure was defined as in-hospital initiation with a discharge prescription.

Among 122 recipients, 116 received empagliflozin and 7 received dapagliflozin (one patient received both sequentially). The predominant dose was 10 mg daily for both agents.

### Outcomes

The primary outcome was 30-day all-cause readmission, defined as any unplanned inpatient admission within 30 days of discharge. Planned readmissions identified by scheduled admission type were excluded. All-cause readmission was selected because it aligns with the CMS HRRP penalty metric, avoids misclassification from inconsistent heart failure coding, and captures pleiotropic SGLT2 inhibitor effects across cardiac, renal, and metabolic pathways. Readmissions were ascertained through the NYU Langone EHR. Out-of-system readmissions were not captured. Secondary outcomes were 90-day all-cause readmission and 30-day all-cause mortality.

### Primary subgroup

The HFrEF subgroup (LVEF ≤ 40%) was the prespecified primary analysis population. This designation was based on Class I guideline evidence [1], the strongest trial evidence base (DAPA-HF, EMPEROR-Reduced, DAPA ACT HF–TIMI 68) [6, 7, 10], and prior observational data showing the largest absolute readmission benefit in this phenotype [11]. The full 438-patient cohort served as a secondary analysis.

### Covariates

Twenty-four pre-treatment covariates were included in the propensity score: age, sex, race/ethnicity, LVEF category, prior heart failure hospitalization within 12 months, hypertension, diabetes mellitus, atrial fibrillation, coronary artery disease, chronic kidney disease, COPD, prior stroke or TIA, admission systolic blood pressure, admission weight, admission eGFR (CKD-EPI 2021), admission hemoglobin, admission creatinine, admission sodium, admission albumin, ICU admission, inotrope use, hospital site, calendar year, and insurance type. All variables temporally preceded treatment decisions.

Discharge GDMT (evidence-based beta-blocker [carvedilol, metoprolol succinate, or bisoprolol], ACEi/ARB/ARNI, MRA) was co-prescribed at the same encounter as SGLT2 inhibitor initiation. We did not include discharge GDMT in the primary propensity model because co-prescribing makes it methodologically inseparable from the exposure in observational data [15]. A sensitivity propensity model adding GDMT (Model B) is reported alongside the primary model (Model A).

### Statistical analysis

Baseline characteristics were compared using standardized mean differences (SMD), with |SMD| >0.20 indicating meaningful imbalance. Stabilized inverse probability of treatment weighting (IPTW) was the primary adjustment [16]. Weights were truncated at the 1st and 99th percentiles.

Sandwich (HC1) standard errors were used throughout. Post-weighting balance was assessed by SMD; |SMD| <0.10 indicated adequate balance. The propensity score C-statistic was 0.88 for Model A and 0.91 for Model B.

Overlap weighting (ATO) served as a sensitivity analysis. Additional sensitivity analyses included restriction to patients with eGFR ≥ 25 mL/min/1.73 m² and propensity score re-estimation within the HFrEF subgroup. The E-value was calculated to assess robustness to unmeasured confounding [17]. Hierarchical logistic regression decomposed confounding sources; this analysis is reported as exploratory only, given events-per-variable constraints in the primary subgroup; results are summarized below and detailed values are available from the corresponding author.

Missing data among propensity score covariates were minimal (admission SBP 1.6%, admission weight 1.8%, admission eGFR 0.2%) and imputed by covariate means. Two-sided *p* < 0.05 was considered significant. Analyses were performed in R version 4.6.0 (WeightIt and survey packages).

## Results

### Cohort

**Fig. 1:**
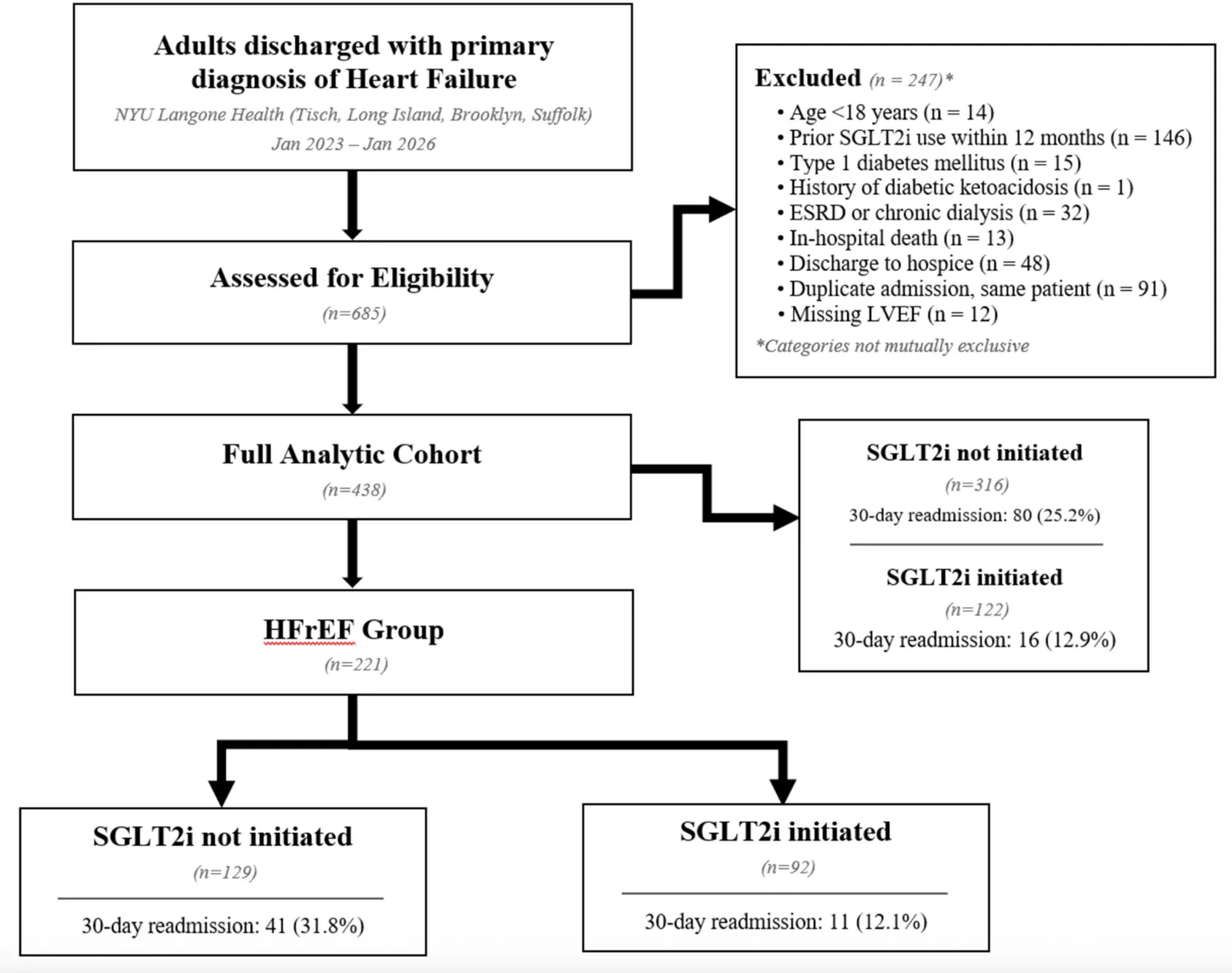
Selection of the study cohort. Of 685 adults assessed for eligibility, 247 were excluded; categories are not mutually exclusive. The full analytic cohort comprised 438 patients (122 SGLT2 inhibitor recipients, 316 controls). The HFrEF subgroup, prespecified as the primary analysis population, included 221 patients (92 SGLT2 inhibitor recipients, 129 controls). Crude 30-day all-cause readmission rates are shown for each group. DKA, diabetic ketoacidosis; DM, diabetes mellitus; ESRD, end-stage renal disease; HFrEF, heart failure with reduced ejection fraction; LVEF, left ventricular ejection fraction; SGLT2i, sodium-glucose cotransporter 2 inhibitor

Of 685 adults assessed for eligibility, 247 were excluded and 438 patients formed the analytic cohort: 122 SGLT2 inhibitor recipients and 316 controls (Fig. 1). The HFrEF subgroup included 221 patients (92 SGLT2 inhibitor, 129 control). Tisch Hospital contributed 61.0% of encounters; Brooklyn, Long Island, and Suffolk contributed 15.5%, 17.8%, and 5.5%, respectively.

### Baseline characteristics

Baseline characteristics are shown in Table 1. SGLT2 inhibitor recipients were younger (60.2 vs. 68.7 years, SMD 0.47), more often male (63.9% vs. 45.9%, SMD 0.37), and had lower LVEF (median 22.5% vs. 50%, SMD 0.87). HFrEF was present in 75.4% of recipients versus 40.8% of controls (SMD 0.75). Recipients had higher admission eGFR (77.6 vs. 68.0 mL/min/1.73 m², SMD 0.41) and lower comorbidity burden across CKD (16.4% vs. 38.9%, SMD 0.52) and CAD (38.5% vs. 64.6%, SMD 0.54).

**Table 1:** Baseline characteristics by SGLT2 inhibitor exposure status

	Overall (*N* = 438)	SGLT2i (*n* = 122)	No SGLT2i (*n* = 316)	SMD
Demographics				
Age, years (mean ± SD)	66.3 ± 18.7	60.2 ± 17.5	68.7 ± 18.7	0.47
Female, n (%)	215 (49.1)	44 (36.1)	171 (54.1)	0.37
Race/ethnicity, n (%)				0.13
White	236 (53.9)	61 (50.0)	175 (55.4)	
Black/African American	102 (23.3)	37 (30.3)	65 (20.6)	
Hispanic/Latino	43 (9.8)	11 (9.0)	32 (10.1)	
Other/Unknown	57 (13.0)	13 (10.7)	44 (13.9)	
Medicare/Medicaid, n (%)	329 (75.1)	81 (66.4)	248 (78.5)	0.23
Calendar year, n (%)				0.09
2023	139 (31.7)	37 (30.3)	102 (32.3)	
2024	138 (31.5)	35 (28.7)	103 (32.6)	
2025	161 (36.8)	50 (41.0)	111 (35.1)	
Hospital site, n (%)				0.17
Tisch (Manhattan)	268 (61.0)	69 (56.6)	199 (62.7)	
Brooklyn	68 (15.5)	30 (24.6)	38 (12.0)	
Long Island (Nassau County)	78 (17.8)	19 (15.6)	59 (18.7)	
Suffolk	24 (5.5)	4 (3.3)	20 (6.3)	
Heart failure phenotype				
LVEF, %, median [IQR]	35 [20–60]	22.5 [15–38]	50 [25–62.5]	0.87
HF phenotype, n (%)				0.75
HFrEF (LVEF ≤ 40%)	221 (50.5)	92 (75.4)	129 (40.8)	
HFmrEF (LVEF 41–49%)	35 (8.0)	11 (9.0)	24 (7.6)	
HFpEF (LVEF ≥ 50%)	182 (41.6)	19 (15.6)	163 (51.6)	
Prior HF hospitalization (12 mo), n (%)	44 (10.0)	5 (4.1)	39 (12.3)	0.30
NT-proBNP, pg/mL, median [IQR]	1024 [415–2083]	1241 [536–2696]	957 [368–1733]	0.27
Comorbidities, n (%)				
Diabetes mellitus	163 (37.2)	54 (44.3)	109 (34.5)	0.20
Hypertension	227 (51.8)	82 (67.2)	145 (45.9)	0.44
Chronic kidney disease	143 (32.6)	20 (16.4)	123 (38.9)	0.52
Atrial fibrillation	204 (46.6)	59 (48.4)	145 (45.9)	0.05
Coronary artery disease	251 (57.3)	47 (38.5)	204 (64.6)	0.54
COPD	96 (21.9)	25 (20.5)	71 (22.5)	0.05
Prior stroke / TIA	113 (25.8)	15 (12.3)	98 (31.0)	0.47
Admission and hospital course				
Admission SBP, mmHg (mean ± SD)	124.6 ± 21.0	125.4 ± 19.4	124.2 ± 21.6	0.06
Admission weight, kg (mean ± SD)	80.60 ± 30.65	91.15 ± 34.10	76.29 ± 28.29	0.47
Admission hemoglobin, g/dL (mean ± SD)	12.14 ± 2.38	12.98 ± 2.25	11.84 ± 2.39	0.49
Admission albumin, g/dL (mean ± SD)	3.61 ± 0.50	3.64 ± 0.44	3.59 ± 0.52	0.10
Admission creatinine, mg/dL (mean ± SD)	1.14 ± 0.54	1.07 ± 0.38	1.17 ± 0.60	0.20
Admission sodium, mEq/L (mean ± SD)	137.46 ± 4.39	137.99 ± 3.75	137.20 ± 4.62	0.19
Admission eGFR, mL/min/1.73 m²	70.7 ± 24.5	77.6 ± 22.5	68.0 ± 24.7	0.41
Length of stay, days, median [IQR]	5 [4–9]	5 [4–9]	5 [3–9]	0.08
ICU admission, n (%)	55 (12.6)	17 (13.9)	38 (12.0)	0.06
Inotrope use, n (%)	71 (16.2)	19 (15.6)	52 (16.5)	0.02
Discharge GDMT, n (%)				
Beta-blocker (evidence-based)ᵃ	181 (41.3)	90 (73.8)	91 (28.8)	**1.01**
ACEi/ARB/ARNI	148 (33.8)	86 (70.5)	62 (19.6)	**1.19**
MRA	145 (33.1)	80 (65.6)	65 (20.6)	**1.02**
Loop diuretic	335 (76.5)	108 (88.5)	227 (71.8)	**0.43**
Discharge clinical stability				
Discharge SBP, mmHg (mean ± SD)	111.5 ± 15.1	106.4 ± 12.3	113.4 ± 15.6	0.50
In-hospital weight change, kgᵇ	3.1 ± 6.9	4.7 ± 7.4	2.5 ± 6.6	0.31
Discharge eGFR, mL/min/1.73 m²	68.5 ± 25.2	75.3 ± 22.4	66.0 ± 25.8	0.38
Discharge disposition, n (%)				
Home or Self-Care	230 (52.5)	83 (68.0)	147 (46.5)	0.45
Home - under care of Home Health Services	146 (33.3)	31 (25.4)	115 (36.4)	0.24
Skilled Nursing Facility	32 (7.3)	7 (5.7)	25 (7.9)	0.09
Acute Rehab Facility	16 (3.7)	1 (0.8)	15 (4.7)	0.24
Hospice Care in Inpatient Facility	10 (2.3)	0 (0)	10 (3.2)	0.26
Other	4 (0.9)	0 (0)	4 (1.3)	0.25

Hospice Care in Inpatient Facility denotes inpatient comfort-focused admission; patients with discharge to external hospice facilities were excluded at the eligibility stage (*n* = 48)

Data presented as mean ± SD, median [IQR], or n (%). SMD, standardized mean difference; bold values > 1.00 indicate marked imbalance. SMDs > 0.20 indicate meaningful imbalance

*ACEi* Angiotensin-converting enzyme inhibitor, *ARB* Angiotensin receptor blocker, *ARNI* Angiotensin receptor-neprilysin inhibitor, *NT-proBNP* N-terminal pro-B-type natriuretic peptide, *COPD* Chronic obstructive pulmonary disease, *eGFR* Estimated glomerular filtration rate, *GDMT* Guideline-directed medical therapy, *HF* Heart failure, *HFmrEF* Heart failure with mildly reduced ejection fraction, *HFpEF* Heart failure with preserved ejection fraction, *HFrEF* Heart failure with reduced ejection fraction, *ICU* Intensive care unit, *IQR* Interquartile range, *LVEF* Left ventricular ejection fraction, *MRA* Mineralocorticoid receptor antagonist, *SBP* Systolic blood pressure, *SD* Standard deviation, *SGLT2i* Sodium-glucose cotransporter 2 inhibitor, *TIA* Transient ischemic attack

ᵃEvidence-based beta-blockers: carvedilol, metoprolol succinate, or bisoprolol

ᵇPositive values indicate weight loss (decongestion) during hospitalization

The most extreme imbalances were in discharge GDMT. Evidence-based beta-blocker use was 73.8% versus 28.8% (SMD 1.01), ACEi/ARB/ARNI use was 70.5% versus 19.6% (SMD 1.19), and MRA use was 65.6% versus 20.6% (SMD 1.02). All three GDMT classes exceeded one standard deviation difference, reflecting that clinicians who initiate SGLT2 inhibitors simultaneously optimize all four pillars of neurohormonal blockade.

Clinical stability variables showed a more nuanced pattern. Admission SBP was similar (125.4 vs. 124.2 mmHg). SGLT2 inhibitor recipients achieved greater in-hospital weight loss (4.7 vs. 2.5 kg). Discharge SBP was lower in recipients (106.4 vs. 113.4 mmHg), reflecting greater decongestion. ICU admission (13.9% vs. 12.0%) and inotrope use (15.6% vs. 16.5%) were balanced.

### Prescribing patterns

In-hospital SGLT2 inhibitor initiation occurred in 122 of 438 patients (27.9%). The rate was 41.6% within HFrEF — a sixfold increase from 6.6% reported in INSIGHT-HF during 2020–2021 [11]. Patients with a heart failure hospitalization in the prior 12 months received SGLT2 inhibitors at 11.4% (5/44) versus 29.7% (117/394) in those without (*p* < 0.001). HFrEF was the strongest positive predictor of prescribing (adjusted OR 5.26, 95% CI 2.76–10.02, *p* < 0.001).

Prior heart failure hospitalization was associated with lower odds of receiving SGLT2 inhibitor (adjusted OR 0.36, 95% CI 0.12–1.05, *p* = 0.061).

### Primary outcome: 30-day all-cause readmission in HFrEF

Among 221 HFrEF patients, 30-day all-cause readmission was 12.1% (11/92) in SGLT2 inhibitor recipients versus 31.8% (41/129) in controls. The crude OR was 0.29 (95% CI 0.14–0.61, *p* = 0.001), an absolute risk difference of 19.7% points.

**Fig. 2:**
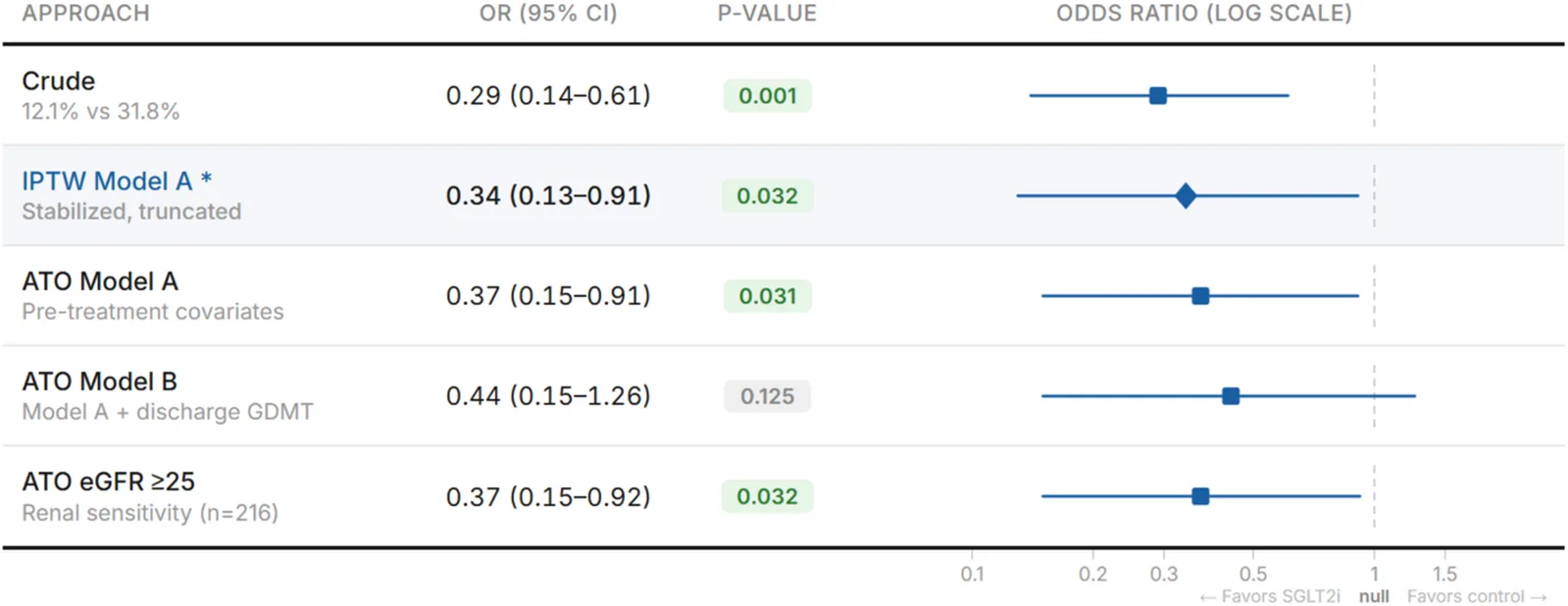
Thirty-day all-cause readmission in the HFrEF subgroup: analytical approaches. Filled squares indicate point estimates; horizontal lines indicate 95% confidence intervals. The primary stabilized IPTW estimate (□) is highlighted in yellow. The dashed vertical reference at OR = 1.0 indicates the null. All five adjusted approaches show point estimates favoring SGLT2 inhibitor initiation; four of five achieve p < 0.05. Point estimates ranged from 0.27 to 0.44.ATO, average treatment effect in the overlap population; CI, confidence interval; eGFR, estimated glomerular filtration rate; GDMT, guideline-directed medical therapy; HFrEF, heart failure with reduced ejection fraction; IPTW, inverse probability of treatment weighting; OR, odds ratio; PS, propensity score

Propensity-weighted analyses are shown in Table 2; Fig. 2. The primary stabilized IPTW estimate was OR 0.34 (95% CI 0.13–0.91, *p* = 0.032). Post-weighting balance was achieved on all major covariates in the full cohort (all |SMD| <0.10). Within the HFrEF subgroup, IPTW did not achieve complete covariate balance; the largest residual SMD was 0.23 for admission albumin (Supplementary Figure, Panel A). ATO weighting achieved near-complete balance (Panel B), and within-HFrEF propensity score re-estimation achieved complete balance (all |SMD| <0.10; see Sensitivity Analyses). The E-value for the primary IPTW point estimate was 5.29; the E-value for the lower confidence bound was 1.42.

**Table 2:** Thirty-day all-cause readmission in the HFrEF subgroup (*N* = 221): propensity-weighted analyses

Analytical approach	OR (95% CI)	*p*-value
Crude (12.1% vs. 31.8%)	0.29 (0.14–0.61)	0.001
Stabilized IPTW, Model A (primary)	**0.34 (0.13–0.91)**	**0.032**
ATO, Model A (pre-treatment covariates)	0.37 (0.15–0.91)	0.031
ATO, Model B (Model A + discharge GDMT)	0.44 (0.15–1.26)	0.125
ATO, eGFR ≥ 25 mL/min/1.73 m² (*n* = 216)	0.37 (0.15–0.92)	0.032
Within-HFrEF propensity score re-estimation	0.27 (0.12–0.63)	0.002

Propensity score Model A included 24 pre-treatment covariates (demographics, heart failure phenotype, comorbidities, admission laboratories and vitals, hospital site, ICU admission, inotrope use, calendar year); C-statistic 0.88. Model B added discharge GDMT (evidence-based beta-blocker, ACEi/ARB/ARNI, MRA); C-statistic 0.91. Stabilized IPTW weights were truncated at the 1st and 99th percentiles. Sandwich (HC1) standard errors throughout. Post-weighting balance achieved |SMD| <0.10 in the full cohort. Within HFrEF, residual covariate imbalance remained after IPTW (largest residual SMD 0.23); see Supplementary Figure. E-value for the primary IPTW point estimate: 5.29; for the lower confidence bound: 1.42

*ATO* Average treatment effect in the overlap population,* CI* Confidence interval, eGFR Estimated glomerular filtration rate,* GDMT* Guideline-directed medical therapy,* HFrEF* Heart failure with reduced ejection fraction,* IPTW* Inverse probability of treatment weighting,* OR* Odds ratio

### Sensitivity analyses

Overlap weighting (ATO Model A) yielded OR 0.37 (95% CI 0.15–0.91, *p* = 0.031). When discharge GDMT was added to the propensity score (Model B), the estimate attenuated to OR 0.44 (95% CI 0.15–1.26, *p* = 0.125). Restriction to patients with eGFR ≥ 25 mL/min/1.73 m² yielded OR 0.37 (95% CI 0.15–0.92, *p* = 0.032). Propensity score re-estimation within the HFrEF subgroup achieved complete covariate balance (all |SMD| <0.10) and yielded OR 0.27 (95% CI 0.12–0.63, *p* = 0.002). All approaches were directionally consistent. Point estimates ranged from 0.27 to 0.44. Ten patients with inpatient comfort-focused care (hospice disposition during the index admission rather than transfer to a hospice facility) remained in the cohort; their exclusion in a sensitivity analysis did not alter the primary finding (IPTW OR 0.38, 95% CI 0.15–0.99, *p* = 0.047).

The interaction test between SGLT2 inhibitor exposure and HFrEF phenotype was not significant (*p* = 0.19). The prespecified HFrEF focus was based on Class I guideline evidence and prior observational data; this result does not confirm differential effects by ejection fraction category. An exploratory hierarchical regression decomposition (available from the corresponding author) suggested that clinical stability parameters contributed approximately 9.3% confounding attenuation, while GDMT was the dominant confounder, although events-per-variable constraints limit interpretation.

### Secondary outcomes and full cohort

Ninety-day all-cause readmission was 22.1% in SGLT2 inhibitor recipients versus 39.6% in controls (crude OR 0.43, 95% CI 0.27–0.70; unadjusted). Thirty-day all-cause mortality was 0.8% versus 3.5% (crude OR 0.23, 95% CI 0.03–1.79, *p* = 0.16), directionally consistent but underpowered. In the full 438-patient cohort, IPTW-adjusted 30-day readmission was directionally consistent but did not reach significance (OR 0.59, 95% CI 0.23–1.49).

## Discussion

In this contemporary US post-2022 guideline cohort of 438 hospitalized heart failure patients, three findings are notable. First, in-hospital SGLT2 inhibitor prescribing in HFrEF reached 41.6%, a sixfold increase from the 6.6% reported in INSIGHT-HF during 2020–2021 [11]. This documents substantial uptake following the guideline update. Second, patients with a heart failure hospitalization in the prior 12 months — the highest-risk group — received SGLT2 inhibitors at only 11.4%, compared with 29.7% in those without recent admission. This gap is an actionable target for hospital quality improvement. Third, in-hospital SGLT2 inhibitor initiation in HFrEF was associated with lower 30-day all-cause readmission across multiple analytical approaches, with propensity-weighted odds ratios ranging from 0.27 to 0.44. Point estimates were directionally consistent across IPTW, overlap weighting, and hierarchical regression, though all share vulnerability to the same unmeasured confounding. The upper end of that range corresponds to the model that accounts for discharge GDMT; the attenuation with GDMT adjustment is addressed in the discussion of confounding below.

The dominant interpretive issue is GDMT co-prescribing. Discharge evidence-based beta-blocker, ACEi/ARB/ARNI, and MRA use were each at least 45% points higher in SGLT2 inhibitor recipients than controls — SGLT2 inhibitor initiation in this cohort was not prescribed in isolation but as part of a broader discharge optimization pattern. The direction of the adjusted association is consistent with the prespecified meta-analysis of EMPULSE, SOLOIST-WHF, and DAPA ACT HF–TIMI 68 (HR 0.71 for cardiovascular death or worsening heart failure; HR 0.57 for all-cause mortality) [10], but the magnitude of our observational estimate exceeds these pooled hazard ratios; direct comparison is limited by differences in endpoint, follow-up duration, and study population. This is largely explained by the co-prescribing pattern just described, and secondarily by the control group’s 31.8% readmission rate, which is higher than national averages. The primary IPTW estimate should therefore be interpreted as an upper bound on any independent SGLT2 inhibitor effect. Yan and colleagues [13] reported a directionally opposite signal — HR 1.58 for all-cause readmission and HR 3.75 for HF-specific readmission — but their cohort included both HFrEF and HFpEF without phenotype stratification, did not adjust for discharge GDMT, and predated the 2022 guideline update.

Adding discharge GDMT to the propensity model (Model B) attenuated the estimate from OR 0.37 to OR 0.44, with the confidence interval crossing the null. This pattern is interpretively important: GDMT was prescribed at the same encounter, so it may function as a co-intervention rather than a pure confounder — adjusting for it could partially remove the mechanism of interest rather than confounding alone. That ambiguity cannot be resolved with observational data and would require a trial with standardized background GDMT. What the attenuation does establish is that the discharge optimization pattern — of which SGLT2 inhibitor initiation was one component — accounts for much of the observed association. Hierarchical regression decomposition reinforces this: clinical stability parameters contributed only 9.3% of confounding attenuation, while GDMT was the dominant confounder. Prior claims-based analyses cited missing clinical variables as a key limitation; those variables are not the primary source of residual confounding here.

The prescribing gap in prior-hospitalized patients — 11.4% versus 29.7% — runs against the clinical gradient. Prior heart failure hospitalization predicts 30-day readmission, yet CHAMP-HF registry data show prior hospitalization rates are inversely related to GDMT dose: higher-acuity patients receive less [18]. STRONG-HF randomized patients hospitalized for acute heart failure to intensive GDMT up-titration initiated before discharge and continued post-discharge versus usual care; 180-day all-cause death or heart failure readmission was 15.2% versus 23.3% (*P* = 0.021), the trial stopped early for efficacy [19]. The population most underserved by current SGLT2 inhibitor prescribing has the strongest existing evidence base for discharge GDMT intensification.

Several limitations apply. The study was conducted at a single academic health system. BMI could not be calculated from available structured data as height was not included in our pre-specified data extraction. Frailty, nutritional status beyond serum albumin, functional status, and activities-of-daily-living measures were not captured in the electronic health record. These variables are plausible confounders — clinicians may preferentially avoid SGLT2 inhibitors in frail or functionally dependent patients who are also at higher readmission risk — and their absence should be interpreted as a source of residual confounding by indication.

Heart failure-specific readmission was not available as a secondary endpoint, as readmission diagnosis codes were not included in our pre-specified data extraction. Safety outcomes including urinary tract infection, genital mycotic infection, diabetic ketoacidosis, acute kidney injury, symptomatic hypotension, and treatment discontinuation were not included in our pre-specified data extraction; while these events are recorded in the Epic EHR, systematic extraction and adjudication was not part of our original analysis plan. Accordingly, this study cannot evaluate the short-term safety profile of inpatient SGLT2 inhibitor initiation, and benefit-risk conclusions should not be drawn from these data alone. Readmission and prior hospitalization ascertainment were limited to the NYU Langone EHR. Out-of-system readmissions were not captured. Because out-of-system readmissions were not captured, 30-day all-cause readmission may have been misclassified. The direction of bias is uncertain and may depend on health-system engagement, insurance, geography, discharge destination, and post-discharge care patterns. The prior heart failure hospitalization rate of 10% likely underestimates the true proportion, as admissions at outside institutions were not captured — this may have affected both the baseline characteristic and its use as a propensity score covariate. Initiation timing during hospitalization was not recorded; early versus late initiation and discharge-only prescribing may reflect different clinical decisions and risk profiles that this study cannot distinguish. The exposure definition captures discharge prescription but not post-discharge adherence or persistence; non-adherence would bias estimates toward the null.

Residual confounding by indication is plausible; the E-value lower confidence bound of 1.42 indicates that an unmeasured confounder of moderate magnitude could shift the confidence interval to include the null. In the HFrEF subgroup, propensity weighting did not achieve complete covariate balance, with residual imbalance in illness-severity markers despite weighting (Supplementary Figure), representing a potential source of residual confounding. The SGLT2 inhibitor group was notably younger than controls (60.2 versus 68.7 years), likely reflecting preferential prescribing in younger, lower-risk patients — the same treatment selection pattern that underlies the GDMT imbalance discussed above. Age-stratified analyses were not feasible given the available event count. Additionally, exclusion of patients without a documented LVEF measurement may have introduced implicit age selection, as LVEF ascertainment rates are lower in older, frailer, and more clinically tenuous patients. The HFrEF cohort is predominantly white (53.3%), drawn from a single urban academic health system, and may not generalize to safety-net, rural, or lower-resource settings, or to the older and frailer patients commonly hospitalized with heart failure at those institutions. Given the modest sample size of 221 HFrEF patients and 52 events, with wide confidence intervals, these findings should be considered hypothesis-generating rather than definitive estimates of effect. Events-per-variable in the HFrEF regression-based analyses was below the conventional threshold of 10 [20], the propensity-weighted analyses, which do not depend on outcome model stability, are therefore the primary estimates for this subgroup. The interaction test between SGLT2 inhibitor exposure and HFrEF phenotype was not significant (*p* = 0.19); the prespecified HFrEF analysis was based on Class I guideline evidence and prior observational data, and the non-significant interaction does not confirm differential effects by ejection fraction category.

## Conclusion

In this contemporary US post-2022 guideline cohort, in-hospital SGLT2 inhibitor prescribing in HFrEF reached 41.6% — a sixfold increase from the pre-guideline era — but remained low in patients with recent heart failure hospitalization. In-hospital SGLT2 inhibitor initiation was associated with lower 30-day all-cause readmission across multiple analytical approaches, directionally consistent with the DAPA ACT HF–TIMI 68 prespecified meta-analysis. The magnitude of the observational association likely reflects concurrent guideline-directed medical therapy optimization rather than the independent effect of SGLT2 inhibitors alone. The prescribing gap in patients with prior heart failure hospitalization is an actionable quality-improvement target. Thirty-day all-cause readmission, aligned with the CMS HRRP metric, is a feasible primary endpoint for future pragmatic trials. Because short-term safety events and post-discharge persistence were not systematically captured, these findings should not be interpreted as establishing the benefit-risk profile of inpatient SGLT2 inhibitor initiation. 

## Supplementary Information

Supplementary Material 1.

Supplementary Material 2.

## Data Availability

The data that support the findings of this study are available from the corresponding author upon reasonable request, subject to NYU Langone Health institutional data sharing policies and applicable HIPAA regulations.
