# Supplementary material for: In-hospital SGLT2 inhibitor initiation, prescribing gaps, and 30-day all-cause readmission in heart failure with reduced ejection fraction: a US post-guideline cohort study
Source: BMC Cardiovasc Disord. 2026 Jun 30;26:888. doi: 10.1186/s12872-026-06184-y (PMC13647711; doi:10.1186/s12872-026-06184-y)
Supplement: Supplementary file 1 [file 12872_2026_6184_MOESM1_ESM.docx]

**STROBE Statement — Checklist of Items for Cohort Studies**

*Supplementary Table. STROBE Checklist for: In-Hospital SGLT2 Inhibitor Initiation, Prescribing Gaps, and 30-Day All-Cause Readmission in Heart Failure With Reduced Ejection Fraction: A US Post-Guideline Cohort Study*

| **Item** | **No.** | **Recommendation** | **Reported on Page/Section** |
| --- | --- | --- | --- |
| ***Title and Abstract*** | | | |
| **Title and abstract** | 1 | (a) Indicate the study's design with a commonly used term in the title or the abstract  (b) Provide in the abstract an informative and balanced summary of what was done and what was found | Title page (cohort study indicated in title)  Abstract (structured: Background, Methods, Results, Conclusions) |
| ***Introduction*** | | | |
| **Background/rationale** | 2 | Explain the scientific background and rationale for the investigation being reported | Introduction, paragraphs 1–2 |
| **Objectives** | 3 | State specific objectives, including any prespecified hypotheses | Introduction, paragraphs 3–4 |
| ***Methods*** | | | |
| **Study design** | 4 | Present key elements of study design early in the paper | Methods: Study design and setting |
| **Setting** | 5 | Describe the setting, locations, and relevant dates, including periods of recruitment, exposure, follow-up, and data collection | Methods: Study design and setting |
| **Participants** | 6 | (a) Give the eligibility criteria, and the sources and methods of selection of participants. Describe methods of follow-up  (b) For matched studies, give matching criteria and number of exposed and unexposed | Methods: Study population; Figure 1 (cohort flow)  (b) Not applicable — propensity-weighted analysis (IPTW), not matched cohort |
| **Variables** | 7 | Clearly define all outcomes, exposures, predictors, potential confounders, and effect modifiers. Give diagnostic criteria, if applicable | Methods: Exposure; Outcomes; Covariates |
| **Data sources / measurement** | 8* | For each variable of interest, give sources of data and details of methods of assessment (measurement). Describe comparability of assessment methods if there is more than one group | Methods: Study design and setting (Epic-based EHR);  Methods: Covariates (CKD-EPI 2021, ICD-10-CM I50.x, transthoracic echocardiography) |
| **Bias** | 9 | Describe any efforts to address potential sources of bias | Methods: Statistical analysis (IPTW with 24 covariates; ATO sensitivity; E-value);  Discussion: Limitations (residual confounding by indication; out-of-system readmissions) |
| **Study size** | 10 | Explain how the study size was arrived at | Methods: Study population (all eligible admissions, Jan 2023–Jan 2026);  Figure 1 (cohort selection flow) |
| **Quantitative variables** | 11 | Explain how quantitative variables were handled in the analyses. If applicable, describe which groupings were chosen and why | Methods: Covariates (LVEF categorized into HFrEF/HFmrEF/HFpEF per 2022 guideline);  Methods: Statistical analysis |
| **Statistical methods** | 12 | (a) Describe all statistical methods, including those used to control for confounding  (b) Describe any methods used to examine subgroups and interactions  (c) Explain how missing data were addressed  (d) If applicable, explain how loss to follow-up was addressed  (e) Describe any sensitivity analyses | (a) Methods: Statistical analysis (stabilized IPTW, 24 covariates)  (b) Methods: Primary subgroup; Statistical analysis (HFrEF prespecified; interaction test reported in Results)  (c) Methods: Statistical analysis (mean imputation; missingness <2%)  (d) Not applicable — administrative readmission ascertainment through EHR  (e) Methods: Statistical analysis (ATO; eGFR ≥25; within-HFrEF re-estimation; E-value) |
| ***Results*** | | | |
| **Participants** | 13* | (a) Report numbers of individuals at each stage of study—eg numbers potentially eligible, examined for eligibility, confirmed eligible, included in the study, completing follow-up, and analysed  (b) Give reasons for non-participation at each stage  (c) Consider use of a flow diagram | (a) Results: Cohort; Figure 1  (b) Figure 1 (exclusion categories with counts)  (c) Figure 1 (cohort flow diagram) |
| **Descriptive data** | 14* | (a) Give characteristics of study participants (eg demographic, clinical, social) and information on exposures and potential confounders  (b) Indicate number of participants with missing data for each variable of interest  (c) Summarise follow-up time (eg, average and total amount) | (a) Results: Baseline characteristics; Table 1  (b) Methods: Statistical analysis (admission SBP 1.6%, weight 1.8%, eGFR 0.2%)  (c) Methods: Outcomes (30-day and 90-day fixed follow-up windows from discharge) |
| **Outcome data** | 15* | Report numbers of outcome events or summary measures over time | Results: Primary outcome (30-day all-cause readmission events with rates)  Results: Secondary outcomes (90-day readmission; 30-day mortality)  Table 2 |
| **Main results** | 16 | (a) Give unadjusted estimates and, if applicable, confounder-adjusted estimates and their precision (eg, 95% confidence interval). Make clear which confounders were adjusted for and why they were included  (b) Report category boundaries when continuous variables were categorized  (c) If relevant, consider translating estimates of relative risk into absolute risk for a meaningful time period | (a) Results: Primary outcome; Table 2 (crude OR, IPTW Model A primary, sensitivity analyses); Methods: Covariates  (b) Methods: Covariates (LVEF ≤40% / 41–49% / ≥50%)  (c) Results: Primary outcome (12.1% vs 31.8%; 19.7 percentage-point absolute difference) |
| **Other analyses** | 17 | Report other analyses done—eg analyses of subgroups and interactions, and sensitivity analyses | Results: Sensitivity analyses (ATO, Model B, eGFR ≥25, within-HFrEF re-estimation; interaction test);  Results: Secondary outcomes and full cohort |
| ***Discussion*** | | | |
| **Key results** | 18 | Summarise key results with reference to study objectives | Discussion, paragraph 1 (three principal findings) |
| **Limitations** | 19 | Discuss limitations of the study, taking into account sources of potential bias or imprecision. Discuss both direction and magnitude of any potential bias | Discussion, limitations paragraph (single-center, out-of-system readmissions, residual confounding, EPV constraints, generalizability) |
| **Interpretation** | 20 | Give a cautious overall interpretation of results considering objectives, limitations, multiplicity of analyses, results from similar studies, and other relevant evidence | Discussion, paragraphs 2–3 (comparison with DAPA ACT HF-TIMI 68 meta-analysis and Yan et al. 2024);  Conclusion (cautious framing of observational magnitude) |
| **Generalisability** | 21 | Discuss the generalisability (external validity) of the study results | Discussion, limitations paragraph (single academic health system; predominantly White cohort; safety-net populations) |
| ***Other Information*** | | | |
| **Funding** | 22 | Give the source of funding and the role of the funders for the present study and, if applicable, for the original study on which the present article is based | Declarations: Funding (no external funding) |

***Reference:*** *von Elm E, Altman DG, Egger M, Pocock SJ, Gøtzsche PC, Vandenbroucke JP; STROBE Initiative. The Strengthening the Reporting of Observational Studies in Epidemiology (STROBE) statement: guidelines for reporting observational studies. Lancet. 2007;370(9596):1453–1457.*
